# Knowledge, attitudes, and beliefs regarding skin cancer among health sciences students in Turkey: A cross-sectional study

**DOI:** 10.1590/1516-3180.2024.0089.13052024

**Published:** 2024-07-15

**Authors:** Esin Sevgi Dogan, Ozden Dedeli Caydam

**Affiliations:** I Ress. Assistant, Department of Nursing, Manisa Celal Bayar University, Manisa, Turkey.; 2 Associated Professor, Department of Nursing, Manisa Celal Bayar University, Manisa, Turkey.

**Keywords:** Skin neoplasms., Attitude of health personnel., Health belief model., Students., Knowledge., Attitude., Skin cancer., Belief., Prevention., Sun protection.

## Abstract

**BACKGROUND:**

Healthcare professionals’ knowledge, attitudes, and beliefs regarding skin cancer are important for reducing the future impact of the disease.

**OBJECTIVE:**

This study evaluated university students’ knowledge, attitudes, and beliefs about skin cancer and examined the variables influencing their attitudes and beliefs about the disease.

**DESIGN AND SETTING:**

This descriptive cross-sectional study was conducted at the Faculty of Health Sciences at Manisa Celal Bayar University, Manisa, Turkey.

**METHOD:**

A total of 960 students participated in this study. Data were collected using the Student Introduction Form, Fitzpatrick Skin Type Scale, Skin Cancer and Sun Knowledge Scale (SCSKS), and Health Belief Model Scale for Skin Cancer (HBMSSC).

**RESULTS:**

The mean SCSKS score of the participants was 14.91 ± 4.23. The mean HBSSC scores of the participants were 23.58 ± 7.79 for perceived susceptibility, 14.79 ± 4.59 for perceived severity, 20.64 ± 6.60 for perceived benefits, 15.93 ± 4.09 for perceived barriers, and 21.78 ± 7.14 for self-efficacy. The mean SCSKS total scores of the university students were significantly and positively correlated with the HBMSSC subdimensions. Gender explained 1.58 of the variance in perceived benefits and 1.65 of the variance in self-efficacy, whereas the SCSKS score explained most other variables.

**CONCLUSION:**

The students’ knowledge of skin cancer and sun protection was moderate. Their attitudes and beliefs regarding skin cancer were unexpected. This study identified students’ knowledge of skin cancer and sun protection as the most important variables for improving their attitudes and beliefs about skin cancer.

## INTRODUCTION

Cancer is the leading cause of death worldwide.^1,2^ However, certain types of cancer can be prevented by avoiding risk factors and using current evidence-based prevention strategies.^3^ Skin cancer is one of the preventable types of cancer.^4^ Skin cancer is becoming more common worldwide, particularly in Turkey.^5,6^ “Malignant melanoma,” the most fatal type of skin cancer, is increasingly common, particularly among young individuals.^7^ Skin cancer is the most prevalent type of cancer among individuals aged 25–29 years and the second most common type of cancer in those aged 15–29 years.^8,9^ Therefore, skin cancer prevention practices should primarily target young individuals.^10^

Conducting visual education campaigns, particularly among young individuals, is recommended to improve prevention practices and sun protection against skin cancer (e.g., sun protection and self-skin examination).^11,12^ Numerous studies have been conducted on skin cancer prevention.^13-15^ However, in Turkey, most studies on skin cancer and sun protection have been conducted on primary and secondary school students,^16-20^ with only limited studies on university students.^21-23^

To develop skin cancer prevention behaviors, people’s knowledge, attitudes, and beliefs about the topic must be evaluated.^13,14^ These knowledge, attitudes, and beliefs can lead to the development of specific strategies tailored to the sociocultural contexts of diverse groups. Increased knowledge and positive attitudes and beliefs can influence skin cancer prevention practices.^24^

Healthcare professionals are crucial in providing consumers with health information.^25^ Future healthcare professionals will play a significant role in preventing skin cancer.^26^ Their knowledge, attitudes, and beliefs regarding this issue are important for reducing the impact of the disease in the future. Therefore, this study evaluated university students’ knowledge, attitudes, and beliefs about skin cancer and examined the variables that predict students’ attitudes and beliefs about the disease.

Although there are many studies on this issue in the literature^27-32^, these studies have typically focused on health professionals, medical students, and nursing students. To the best of our knowledge, no such study has been conducted on health science, midwifery, nursing, or social work students. The results of this study will be useful for future research, providing valuable insights into the implementation of skin cancer prevention practices aimed at health science students.

## OBJECTIVE

This study aimed to evaluate health science students’ knowledge, attitudes, and beliefs about skin cancer and to examine the variables influencing their attitudes and beliefs about the disease.

### Research questions

1. What do health science students know about skin cancer and sun protection?
2. What are the attitudes and beliefs of university students about skin cancer?
3. What variables influence health science students’ attitudes and beliefs about skin cancer?

## METHODS

This cross-sectional study included 960 health science students. It was conducted between March and July 2023 at the university’s Faculty of Health Sciences in the nursing, midwifery, social work, physical therapy, and rehabilitation departments. No sampling method was used in this study, resulting in a participation rate of 61.34%.

The inclusion criteria were participants aged 18 years or older who agreed to participate. Students who chose to leave the study or did not complete the forms were excluded.

### Instruments

Based on the literature, the Information Form for Students included nine questions on demographic data (age and gender), sun exposure, and sunburn history.^18,20-23,33,34^

### The Fitzpatrick Skin Type Scale

The Fitzpatrick skin type scale was used as a classification scheme. The scale was divided into six categories according to the skin’s susceptibility to sunburn, indicating that the risk of developing skin cancer reduces from skin types 1 to 6. Skin types 1 and 2 have a high risk of developing skin cancer, skin types 3 and 4 have a moderate risk, and skin types 5 and 6 have a low risk.^35^

### Skin Cancer and Sun Knowledge Scale

The Skin Cancer and Sun Knowledge Scale (SCSKS) was developed for young adults (aged 18 to 26).^36^ The scale comprises 25 items that evaluate skin cancer and sun knowledge. The domains include sun production, tanning, skin cancer risk factors, skin cancer prevention, and skin cancer symptoms. The total score ranges from 0 to 25, with a high score on this scale indicating a high level of knowledge.^34,36^ The Turkish validity and reliability of the scale were evaluated among nursing students in a previous study, with an internal consistency reliability coefficient (KR-20) of 0.51.^34^ In this study, it was determined to be 0.52.

### Health Belief Model Scale in Skin Cancer

The Health Belief Model Scale in Skin Cancer (HBMSSC) was developed by Dogan and Caydam (2021) for university students.^37^ The scale comprises 26 items and five subdimensions: perceived susceptibility, perceived benefit, perceived severity, perceived barriers, and self-efficacy. Each item on the scale received was scored as follows: 5 = *strongly agree*, 4 = *agree*, 3 = *neutral*, 2 = *strongly disagree*, or 1 = *disagree*. The subdimension “perceived barriers” is reverse coded, and the HBMSSC does not have a total score. Higher scores on the subdimensions indicate higher perceived susceptibility, perceived benefits, perceived severity, and self-efficacy. The total Cronbach’s α coefficient of the HBMSSC is 0.86, while for the subdimensions, it is 0.89, 0.79, 0.77, 0.65, and 0.86, respectively.^37^ In this study, Cronbach’s α coefficients were 0.97, 0.98, 0.93, 0.94, 0.89, and 0.97, respectively.

### Data Collection

The research data were collected online. Data collection forms were distributed to the students’ class WhatsApp groups via Google Forms. The participants signed an informed consent form if they wished to participate in the study.

### Statistical Analysis

The analysis was performed using Statistical Package for the Social Sciences (SPSS) V15. The suitability of the data for a normal distribution was determined by evaluating the skewness coefficient. The data showed a normal distribution as the coefficient of skewness was between +1 and −1.^38^ Quantitative variables are presented as mean, standard deviation, minimum, and maximum, while qualitative variables are presented as numbers and percentages. Differences between groups were evaluated using the t-test for independent groups and analysis of variance. The homogeneity of variance was determined using Levene’s test. Tukey’s test was used to determine which group caused a difference in three or more groups if the variances followed a homogeneous distribution, whereas Tamhane’s *T* ^2^ test was used if the variances were not equally distributed. Relationships between variables were examined using Pearson’s correlation analysis. After univariate analysis, multivariate regression analysis was used to identify significant variables. Before using the multiple regression model, the relationships between the independent variables in the model were analyzed, and the variables to be included in the model were selected. Statistical significance was set at p < 0.05.

### Ethical Considerations

To conduct this study, ethics committee approval (Manisa Celal Bayar University’s Health Sciences Ethics Committee: 04/01/2023-1653) and institutional permissions (Manisa Celal Bayar University’s Faculty of Health Sciences Deanship-11.01.2023-E-64031256-605.99-465123) required for the conduct of the study were obtained. Students who agreed to participate in the study were informed about its purpose and scope. This study was conducted in accordance with the principles of the Declaration of Helsinki.

## RESULTS

### Descriptive statistics

The study included 84.2% female students, with 44.9% studying nursing and 27.8% in their third year. Among the students, 46.1% had dark brown hair, 72.1% had brown eyes, 42.2% had light skin, and 25.1% had type II skin (**Table 1**).

**Table 1:** Sociodemographic characteristics and skin types of participants (n = 960)

Age (year)	± SD	Min−Max
	ߓ21.28 ± 1.99	18–35
**Gender**	*n*	*%*
Female	808	84.2
Male	152	15.8
**Training department**		
Nursing	431	44.9
Midwifery	227	23.6
Physical therapy and rehabilitation	219	22.8
Social work	83	8.6
**Class**		
First	226	23.5
Second	227	23.6
Third	267	27.8
Fourth	240	25.0
**Hair color**		
Fair	99	10.3
Light brown	182	19.0
Dark brown	443	46.1
Black	236	24.6
**Eye color**		
Blue/green	83	8.6
Hazel	115	12.0
Brown	692	72.1
Black	70	7.3
**Skin color**		
Fair	405	42.2
Auburn/light brown	293	30.5
Brown/brunette	262	27.3
**Skin type**		
Type I	89	9.3
Type II	241	25.1
Type III	233	24.3
Type IV	206	21.5
Type V	191	19.9
**History of sunburn in the last one year**		
No	457	47.6
Once	234	24.4
Twice	148	15.4
Three times or more	121	12.6
**Region living with family**		
Aegean	528	55.0
Mediterranean	107	11.1
Southeastern Anatolian	78	8.1
Eastern Anatolian	54	5.6
Inner Anatolian	66	6.9
Black Sea	28	2.9
Marmara	99	10.3

Max = maximum; min = minimum; SD = standard deviation.

### Skin cancer and sun knowledge

The mean SCSKS score of the students was 14.91. The SCSKS scores varied significantly depending on gender, class, department, hair color, and skin color (**Table 2**).

**Table 2:** Assessment of Skin Cancer and Sun Knowledge Scale scores and Health Belief Model Scale scores among students (n = 960)

Participants (n = 960)	SCSKS	PSus	PSev	PBen	PBar	SE
	14.91 ± 3.02	23.58 ± 7.79	14.79 ± 4.59	20.64 ± 6.60	15.93 ± 4.09	21.78 ± 7.14
**Gender**						
Female (*n*=808)	15.10 ± 2.98	24.12 ± 7.35	15.11 ± 4.35	21.11 ± 6.26	-	22.34 ± 6.83
Male (*n* = 152)	13.91 ± 3.07	20.66 ± 9.33	13.13 ± 5.47	18.16 ± 7.74	-	18.83 ± 8.02
*t/p*	**4.528/<0.000**	**5.085/0.000**	**4.951/0.000**	**5.108/0.000**	-	**5.645/0.000**
**Training department**						
Nursing (*n* = 431)^a^	14.96 ± 3.13	23.34 ± 7.87	14.72 ± 4.62	20.60 ± 6.71	15.32 ± 4.56	21.60 ± 6.98
Midwifery (*n* = 227)^b^	14.55 ± 2.82	25.58 ± 6.29	15.72 ± 3.83	22.51 ± 5.26	16.69 ± 3.69	23.70 ± 6.03
Physical therapy and rehabilitation (*n* = 219)^c^	14.91 ± 2.96	22.02 ± 8.51	13.76 ± 4.97	19.02 ± 6.96	16.11 ± 3.24	20.40 ± 7.91
Social work (*n* = 83)^d^	15.62 ± 2.99	23.39 ± 8.07	15.39 ± 4.91	20.02 ± 7.20	16.54 ± 3.21	21.09 ± 7.65
*F/p*	**2.636/0.049** a = b = c, d > b^*^	**8.252/0.000** a = c = d, b = d, b *>* a, b *>* c^*^	**7.356/0.000** a = c = d, b = d, b *>* a, b *>* c^*^	**10.998/0.000** a = d, c = d, b *>* a *>* c, b *>* d^*^	**6.955/0.000** a = c, b = c = d, b *>* a, d *>* a^*^	**8.740/0.000** a=c=d, b>a, b>c, b>d^*^
**Class**						
First (*n* = 226)^a^	14.28 ± 3.04	22.35 ± 8.13	14.37 ± 4.86	19.95 ± 7.08	-	20.71 ± 7.65
Second (*n* = 227)^b^	14.65 ± 2.98	23.49 ± 7.63	14.75 ± 4.59	20.64 ± 6.62	-	21.49 ± 6.85
Third (*n* = 267)^c^	15.22 ± 3.03	24.52 ± 7.74	15.13 ± 4.46	21.18 ± 6.33	-	22.76 ± 7.10
Fourth (*n* = 240)^d^	15.42 ± 2.89	23.75 ± 7.57	14.87 ± 4.49	20.69 ± 6.40	-	21.97 ± 6.84
*F/p*	**7.204/<0.000** c = d > a = b^*^	**3.222/0.022** a = b = d, c = d, b = b, c *>* a^**^	1.148/0.329	1.436/0.231	-	**3.569/0.014** a = b = d, c = d, c *>* a^**^
**Hair color**						
Fair (*n* = 99)^a^	15.16 ± 2.86	23.95 ± 7.82	15.40 ± 4.66	20.20 ± 6.45	-	21.90 ± 6.97
Light brown (*n* = 182)^b^	15.38 ± 3.34	23.59 ± 7.59	14.77 ± 4.47	20.79 ± 6.51	-	22.39 ± 7.30
Dark brown (*n* = 443)^c^	14.91 ± 2.92	24.19 ± 7.33	15.09 ± 4.29	21.21 ± 6.25	-	22.28 ± 6.73
Black (*n* = 236)^d^	14.46 ± 2.96	22.26 ± 8.63	14.01 ± 5.13	19.65 ± 7.26	-	20.32 ± 7.67
*F/p*	**3.482/0.015** a = b, a = c, a = d, c = d, b > c, b > d^**^	**3.234/0.022** a = b = c, a = d, b = d, c *>* d^*^	**3.546/0.014** a = b = c, a = d, a = c, b = d, c *>* d^*^	**3.076/0.027** a = b = c, a = d, a = c, b = d, c *>* d^*^	-	**4.537/0.004** a = b = c, a = d, a = c, b = d, c *>* d^*^
**Skin color**						
Fair (*n* = 405)^a^	15.30 ± 2.98	-	-	-	-	-
Auburn/light brown (*n* = 293)^b^	14.67 ± 2.98	-	-	-	-	-
Brown/brunette (*n* = 262)^c^	14.58 ± 3.05	-	-	-	-	-
*F/p*	**6.056/0.002** a > b = c^**^	-	-	-	-	-
**Region living with family**						
Mediterranean (*n* = 107)^a^	-	23.65 ± 7.58	14.68 ± 4.58	21.10 ± 6.66	-	22.25 ± 7.10
Eastern Anatolian (*n* = 54)^b^	-	19.24 ± 9.58	12.72 ± 5.74	16.62 ± 7.49		17.48 ± 8.27
Aegean (*n* = 528)^c^	-	24.46 ± 7.28	15.28 ± 4.25	21.20 ± 6.30	-	22.54 ± 6.64
Southeastern Anatolian (*n* = 78)^d^	-	22.88 ± 7.89	14.47 ± 4.52	21.07 ± 6.42	-	20.92 ± 7.23
Inner Anatolian (*n* = 66)^e^	-	22.22 ± 8.27	13.78 ± 5.13	19.95 ± 6.56	-	20.42 ± 7.79
Black Sea (*n* = 28)^f^	-	20.42 ± 9.34	12.39 ± 5.23	19.21 ± 7.91	-	19.96 ± 8.71
Marmara (*n* = 99)^g^	-	23.43 ± 7.68	15.06 ± 4.67	19.85 ± 6.62	-	21.61 ± 7.11
*F/p*	-	**5.275/0.000** a = b = d = e = f = g, a = c = d = e = f = g, c *>* b^*^	**4.884/0.000** a = b = d = e = f = g, a = c = d = e = f = g, c *>* b^**^	**4.783/0.000** a = b = d = e = f = g, a = c = d = e = f = g, c *>* b^*^	-	**5.399/0.000** a = b = d = e = f = g, a = c = d = e = f = g, c *>* b^*^

*t*, independent t-test; F, one-way analysis of variance; SCSKS, Skin Cancer Sun Knowledge Scale; Pbar, perceived barriers; PBen, perceived benefits; PSev, perceived severity; PSus, perceived susceptibility; SE, self-efficacy; Significance, p < 0.05; ^*^Tamhane’s *T* ^2^ test, ^**^Tukey’s test.

### Attitudes and beliefs about skin cancer

The attitudes and beliefs about skin cancer are presented in **Table 2**. The mean HBSSC scores were 23.58 ± 7.79 for perceived susceptibility, 14.79 ± 4.59 for perceived severity, 20.64 ± 6.60 for perceived benefits, 15.93 ± 4.09 for perceived barriers, and 21.78 ± 7.14 for self-efficacy.

The mean perceived susceptibility scores were significantly higher in female participants studying in the midwifery department, third grade students, those with dark brown hair, and those living in the Aegean Region with their families.

The mean perceived severity scores were significantly higher among female students in the midwifery department, those with fair hair color, and those living in the Aegean Region with their families.

The mean perceived benefits scores were significantly higher in female participants studying in the midwifery department, those with dark brown hair, and those living in the Aegean Region with their families.

The mean perceived barrier score was significantly higher among those studying in the midwifery department.

The mean self-efficacy scores of female participants studying in the midwifery department, those with light brown hair, and those living in the Aegean Region with their families were significantly higher.

### Relationship between knowledge about skin cancer and attitudes and beliefs about the disease

A significant positive correlation was observed between the mean SCSKS total score of the students and the HBMSSC sub-dimensions: perceived susceptibility (r = 0.193, *p* < 0.001), perceived severity (r = 0.176, *p* < 0.001), perceived benefits (r = 0.130, *p* < 0.001), perceived barriers (r = 0.120, *p* < 0.001), and self-efficacy scores (r = 0.167, *p* < 0.001) (**Table 3**).

**Table 3:** Mean SCSKS and HBMSSC score correlations (*n =* 960)

	PSus	PSev	PBen	PBar	SE
	*r/p*				
SP-SS	**0.083/0.010**	0.063/0.052	0.046/0.155	0.035/0.275	**0.075/0.044**
T-SS	**0.105/0.001**	**0.121/< 0.001**	0.060/0.062	**0.090/0.005**	**0.095/0.003**
SCP-SS	**0.165/< 0.001**	**0.119/< 0.001**	**0.105/0.001**	−0.047/0.148	**0.120/< 0.001**
SCRF-SS	**0.186/< 0.001**	**0.139/< 0.001**	**0.154/0.001**	**0.092/0.004**	**0.163/< 0.001**
SSC-SS	**0.087/0.007**	**0.085/0.009**	**0.840/0.009**	**0.076/0.018**	**0.090/0.005**
SCSKS	**0.193/< 0.001**	**0.176/< 0.001**	**0.130/< 0.001**	**0.120/< 0.001**	**0.167/< 0.001**

Pbar, perceived barriers; PBen, perceived benefits; PSev, perceived severity; PSus, perceived susceptibility; *r*, Pearson correlation analysis; SCP-SS = Skin Cancer Prevention Subscale; SCRF-SS = Skin Cancer Risk Factors Subscale; SCSKS = Skin Cancer and Sun Knowledge Scale; SP-SS = Sun Production Subscale; SE, self-efficacy; SSC-SS = Symptoms of Skin Cancer Subscale; T-SS = Tanning. p < 0.05.

**Table 4** presents the raw and standardized regression coefficients for each analysis step.

**Table 4:** Variables predicting students’ attitudes and beliefs about skin cancer (*n* = 960)

Model 1	PSus				PSev				PBen				PBar				SE			
	*R* = 0.190/*R* ^2^ = 0.036 *F* = 7.167/*p* = 0.000)				*R* = 0.167/*R* ^2^ = 0.028 *F* = 6.830/*p* = 0.000				*R* = 0.184/*R* ^2^ = 0.034 *F* = 8.375/*p* = 0.000				—				*R* = 0.212/*R* ^2^ = 0.045 *F* = 8.839/*p* = 0.000			
	*B*	*SE*	*β*	*t/p*	*B*	*SE*	*β*	*t/p*	*B*	*SE*	*β*	*t/p*	*B*	*SE*	*β*	*t/p*	*B*	*SE*	*β*	*t/p*
Constant	27.567	1.412	—	19.529/ < 0.001	18.052	0.761	—	23.735/ < 0.001	25.223	1.089	—	23.165/ < 0.001	—	—	—	—	26.172	1.285	—	20.365/ 0.001
Gender	−3.589	0.700	−0.168	**−5.127/** **< 0.001**	−1.909	0.414	−0.152	**−4.615/** **< 0.001**	−3.119	0.592	−0.172	**−5.268/** **< 0.001**	—	—	—	—	−3.608	0.639	−0.185	**−5.650/** **< 0.001**
Department	−0.322	0.252	−0.042	−1.279/ 0.201	−0.157	0.149	−0.035	−1.055/ 0.292	−0.501	0.213	−0.077	**−2.352/** **0.019**	—	—	—	—	−0.339	0.230	−0.057	−1.739/ 0.082
Class	0.587	0.226	0.083	**2.603/** **0.009**	—	—	—	—	—	—	—	—	—	—	—	—	0.561	0.206	0.087	**2.731/** **0.006**
Hair color	−0.084	0.225	−0.012	−0.373/ 0.709	−0.162	0.132	−0.040	−1.222/ 0.222	0.086	0.190	−0.015	−0.454/ 0.650	—	—	—	—	−0.138	0.204	−0.022	−0.605/ 0.500
Living region	−0.111	0.158	−0.023	−0.704 /0.481	−0.037	0.093	−0.013	−0.402/ 0.688	−0.091	0.134	−0.022	−0.678/ 0.498	—	—	—	—	−0.100	0.144	−0.022	−0.695/ 0.487
Model 2	*R* = **0.249/** *R* ^2^ = **0.062** *F* = **10.486/** *p* = **0.000**				*R* = **0.227/** *R* ^2^ = **0.052** *F* = **10.390/** *p* = **0.000**				*R* = **0.215/** *R* ^2^ = **0.046** *F* = **9.208/** *p* = **0.000**				*R* = **0.157/** *R* ^2^ = **0.025** *F* = **12.025/** *p* = **0.000**				*R* = **0.249/** *R* ^2^ = **0.062** *F* = **10.478/** *p* = **0.000**			
Constant	21.002	1.895	—	11.084/ <0.001	14.112	1.101	—	12.817/ <0.001	21.183	1.586	—	13.357/ <0.001	12.834	0.684	—	18.753/ <0.001	21.268	1.734	−	12.266/ <0.001
Gender	−3.085	0.698	−0.145	**−4.421/** **< 0.001**	−1.649	0.412	−0.131	**−4.001/** **< 0.001**	−2.852	0.594	−0.158	**−4.805/** **< 0.001**	—	—	—	—	−3.231	0.639	−0.165	**−5.053/** **< 0.001**
Department	−0.356	0.249	−0.046	−1.433/ 0.152	−0.171	0.147	−0.038	−1.162/ 0.246	−0.516	0.212	−0.079	**−2.433/** **0.015**	0.399	0.127	0.100	**3.142/** **0.002**	0.427	0.206	0.066	**2.069/** **0.039**
Class	0.407	0.225	0.058	1.805/ 0.071	—	—	—	—	—	—	—	—	—	—	—	—	−0.425	0.228	−0.060	−1.867/ 0.062
Hair color	−0.039	0.222	−0.006	−0.176/ 0.860	−0.131	0.131	−0.033	−0.101/ 0.317	0.118	0.189	0.020	0.623/ 0.533	—	—	—	—	−0.105	0.203	−0.017	−0.519/ 0.604
Living region	−0.107	0.156	−0.022	−0.686/ 0.493	−0.037	0.092	−0.013	−0.396 /0.692	−0.090	0.133	−0.022	−0.675/ 0.500	—	—	—	—	−0.097	0.142	−0.022	−0.680/ 0.497
SCSKS	0.424	0.083	0.164	**5.112/** **< 0.001**	0.238	0.049	0.156	**4.986/** **< 0.001**	0.244	0.070	0.112	**3.486/** **< 0.001**	0.155	0.042	0.117	**3.659/** **< 0.001**	0.317	0.076	0.134	**4.171/** **< 0.001**

Pbar = perceived barriers; PBen = perceived benefits; PSev = perceived severity; PSus = perceived susceptibility; SE = self-efficacy; *SE* = standard error; SCSKS = Skin Cancer and Sun Knowledge Scale.

The SCSKS score explained 1.64, 1.12, 1.56, 1.17, and 1.34 of the variances in perceived susceptibility, perceived severity, perceived benefits, perceived barriers, and self-efficacy, respectively, after controlling for other variables.

Regarding perceived susceptibility, severity, and barriers, gender and SCSKS scores were significant in the final model (Model 2). Gender, training department, and SCSKS scores were significant for perceived benefits and self-efficacy. The training department, SCSKS score, and effective variables for perceived barriers were evaluated. Gender explained 1.58 of the variance in perceived benefits and 1.65 of the variance in self-efficacy, while the SCSKS score explained the majority of the remaining variables.

## DISCUSSION

### Skin Cancer and Sun Knowledge Scale

The mean SCSKS score of the participants was 14.91, indicating moderate knowledge (4.23 ± 1.08), with scores ranging between 0 and 25. Compared to other studies, such as Kasar et al., which reported a mean SCSKS score of 13.64 ± 2.91 for nursing students,^39^ a study comprising medical students demonstrated higher knowledge levels.^40^ However, this knowledge level was not as high as expected, especially considering the importance of health-related professions in educating the public about skin cancer. These results underscore the need for improved education on skin cancer for health science students in Turkey.

This study also identified that students training in the midwifery department and first-year students have lower SCSKS scores. This could be attributed to the limited exposure of first-year students to health education. However, the low scores among midwifery students, predominantly female, were unexpected. Given their role in providing health services and home health training, enhancing skin cancer education in midwifery programs is essential.^41^ The average score of female students was higher than that of male students. Midwives, similar to nurses, play a crucial in providing preventive health services to society and home health training.^42^ Therefore, these results highlight the importance of improving the education of midwifery students in Turkey, suggesting the need for more information on skin cancer in midwifery education programs.

Findings regarding the relationship between skin color and knowledge are inconclusive. While some studies found no difference based on hair color,^39,41,43^ others suggested that individuals with light-colored hair tend to have more knowledge of skin cancer.^44,45^ This study found that knowledge was higher among students with lighter skin. However, these differences may be influenced by the geographical region of the study.

Black-haired students obtained lower knowledge scores, although findings on the relationship between hair color and knowledge are mixed. While the results of this study are comparable with those of other studies,^41,45^ they contradict others.^39^ For instance, sensitivity to skin cancer risk factors, such as light hair, skin, and eye color, is well-documented.^46-47^ Despite many participants having dark brown or black hair, typically considered advantageous for the treatment of skin cancer, black-haired students scored lower on skin cancer knowledge. These findings suggest a need for further research. Perhaps individuals with dark brown or black hair could benefit from information on the risks of skin cancer in their demographic.

Additionally, the mean DKGBÖ test scores of female students were significantly higher than those of male students. These results are consistent with those of previous research.^34,39,41,48^ The results could be attributed to female students’ heightened sensitivity to aesthetic concerns and body image, which may make them more receptive to such topics. Furthermore, the higher perceived susceptibility scores among female students further support this notion.

### Attitudes and beliefs about skin cancer

Only one study has utilized this scale for university students,^37^ thus the study results were compared with those of other studies.

The students’ mean score for perceived susceptibility was 23.58 ± 7.79. Similar to a study conducted on university students,^37^ the scores were positive. However, these results differ from those of a study involving medical students,^49^ where the mean perceived susceptibility score was low. Therefore, we hypothesized that enhancing students’ skin cancer risk education would increase their perceived susceptibility levels.^50^

In the Health Belief Model, perceived severity influences perceived disease threat, thereby increasing the likelihood of preventive action.^50^ The students’ perceived severity was moderate. The study results are comparable to those of medical students,^49^ but higher than those of a study conducted on university students.^37^ This difference may be attributed to the level of knowledge among individuals who keep up with health-related developments. Nonetheless, we anticipate an improvement in this regard. Moreover, this evaluation may indicate that many non-melanoma skin cancers are low-risk and easy to manage, whereas melanoma can be fatal in some cases.^51^

Skin cancer interventions primarily focus on community or individual campaigns.^52^ Visual materials, such as ultraviolet (UV) photography, have been used to evaluate and influence skin cancer protection behaviors among university students.^53-55^ A systematic review^53^ found that UV photography significantly increasesd the perceived severity of photoaging. Furthermore, the authors recommended the use of UV photography and associated educational materials to enhance students’ sun protection behavior.^53^ Therefore, the findings suggest the need for alternative educational methods to enhance students’ perceived severity.

Contrary to the literature,^49^ the mean perceived benefit scores of the students in this study were lower and moderate. These results were unexpected, as we anticipated greater perceived benefits. This discrepancy may be attributed to the average level of skin cancer knowledge and sun exposure awareness in our research sample. Therefore, it is crucial to reflect on these findings when educating health science students. The findings demonstrate that alternative educational methods should be employed to enhance students’ perceived benefits, as health professionals play a critical role in counseling patients on skin cancer and sun-protective behaviors.

The mean perceived barrier scores of the students in this study were positive, consistent with previous studies.^37,49^ These findings indicate that perceived severity, benefits, and self-efficacy can be influenced. Another study on medical students identified a high level of knowledge about skin cancer but inadequate skin self-examination and sun protection behavior, primarily due to a lack of evaluation.^30^

Similar to the literature,^37,49^ students’ mean self-efficacy scores were moderate. However, we expected students who had received health-related education to demonstrate better self-efficacy. Self-efficacy is particularly important for the development of healthy lifestyle behaviors. Our findings suggest that alternative education programs could significantly impact attitudes and beliefs about skin cancer among health science students. Visual educational materials such as brochures, videos, and PowerPoint presentations have been shown to enhance self-efficacy.^20,56^ For instance, an educational intervention supported by visual materials increased skin self-examination behaviors among nursing students in Turkey.^57^

### Relationship between knowledge, attitude, and belief

Gender and SCSKS scores were significant in the final model for perceived susceptibility and severity. Gender, training department, and SCSKS scores were significant for perceived benefits and self-efficacy. The training department, SCSKS score, and effective variables for perceived barriers were evaluated. Gender explained 1.58 of the variance for perceived benefits and 1.65 of the variance for self-efficacy, while the SCSKS score accounted for most of the variance for other variables.

The higher mean scores for perceived benefits and self-efficacy among female students in this study were expected. Female students, being more sensitive to aesthetic concerns and body image, may also be more attentive to the subject and better at self-monitoring. However, most students in this study were female and had only moderate skin cancer and sun protection knowledge, we consider skin cancer and sun protection knowledge to be significant variables in attitudes and beliefs regarding skin cancer.

Although the present study reveals important findings, it has several limitations. First, not all students participated because the study was voluntary. Second, the results may not be generalizable to all health science students as the sample included only health science students from one faculty. Third, the results were based on individual reports. Nonetheless, we believe that the data collection tools were effective in evaluating skin cancer and sun protection knowledge, attitudes, and beliefs regarding skin cancer.

## CONCLUSION

This study revealed a moderate level of skin cancer and sun protection knowledge among health science students, however, their attitudes and beliefs regarding skin cancer were not as expected. While their perceived susceptibility and barriers to skin cancer were positive, their perceived severity, perceived benefits, and self-efficacy were moderate. Furthermore, female gender was a significant factor for perceived benefits and self-efficacy, while skin cancer and sun protection knowledge were significant variables for perceived susceptibility, severity, and barriers. These findings underscore the importance of a comprehensive educational approach to enhance skin cancer attitudes and beliefs among health science students, thereby fostering behavioral changes and promoting skin cancer protection. Effective training programs are crucial for the health and well-being of our study population and the patients they will serve as future health professionals.
